# Additive and supra-additive cytotoxicity of cisplatin-taxane combinations in ovarian carcinoma cell lines

**DOI:** 10.1038/sj.bjc.6690046

**Published:** 1999-01

**Authors:** P Engblom, V Rantanen, J Kulmala, H Helenius, S Grènman

**Affiliations:** Department of Obstetrics and Gynecology, Turku University Central Hospital, Turku, FIN-20520, Finland; Department of Medical Biochemistry, University of Turku, Turku, FIN-20520, Finland; Department of Oncology and Radiotherapy, Turku University Central Hospital, Turku, FIN-20520, Finland; Department of Medical Biostatistics, University of Turku, Turku, FIN-20520, Finland

**Keywords:** taxanes, cisplatin, interaction, chemosensitivity, ovarian carcinoma

## Abstract

The purpose of this study was to compare the growth-inhibitory effect of cisplatin–paclitaxel with that obtained with a cisplatin–docetaxel combination and to assess the type of interaction. Concomitant use of taxanes and cisplatin was studied in seven human ovarian carcinoma cell lines, using the 96-well plate clonogenic assay. Chemosensitivity was expressed in terms of IC_50_ values, the drug concentration causing 50% inhibition of clonogenic survival. The type of interaction was studied using the area under the survival curve ratios (AUC ratios) obtained by numerical integration. Comparison of the AUC ratio and the surviving fraction (SF) value after taxane alone was made using Student's *t*-test. The influence of the drug concentration was tested by one-way analysis of variance (Anova). A supra-additive or additive effect was seen when seven ovarian carcinoma cell lines were exposed to paclitaxel or docetaxel concomitantly with cisplatin. A supra-additive effect was found in four cell lines (UT-OC-3, UT-OC-4, UT-OC-5 and SK-OV-3) after simultaneous use of cisplatin with all docetaxel concentrations tested, and in two cell lines (UT-OC-4 and SK-OV-3) when cisplatin was used concomitantly with paclitaxel. A more pronounced supra-additive effect was seen with the combination of cisplatin and docetaxel. The degree of supra-additivity was dose dependent, with increasing synergy after a higher taxane dose. The data obtained in this study suggest that a supra-additive or additive effect can be achieved in ovarian carcinoma with the concomitant use of cisplatin and a taxane. © 1999 Cancer Research Campaign


*British Journal of Cancer* (1999) **79**(2), 286–292
© 1999 Cancer Research Campaign

### Additive and supra-additive cytotoxicity of cisplatin–taxane combinations in ovarian carcinoma cell lines

**P Engblom[1,2], V Rantanen[1,2], J Kulmala[3], H Helenius[4] and S Grènman[1,2]**

[1]Department of Obstetrics and Gynecology, Turku University Central Hospital, FIN-20520 Turku, Finland; [2]Department of Medical Biochemistry, University of Turku, FIN-20520 Turku, Finland; [3]Department of Oncology and Radiotherapy, Turku University Central Hospital, FIN-20520 Turku, Finland; [4]Department of Medical Biostatistics, University of Turku, FIN-20520 Turku, Finland

**Summary** The purpose of this study was to compare the growth-inhibitory effect of cisplatin–paclitaxel with that obtained with a cisplatin–docetaxel combination and to assess the type of interaction. Concomitant use of taxanes and cisplatin was studied in seven human ovarian carcinoma cell lines, using the 96-well plate clonogenic assay. Chemosensitivity was expressed in terms of $IC_{50}$ values, the drug concentration causing 50% inhibition of clonogenic survival. The type of interaction was studied using the area under the survival curve ratios (AUC ratios) obtained by numerical integration. Comparison of the AUC ratio and the surviving fraction (SF) value after taxane alone was made using Student's *t*-test. The influence of the drug concentration was tested by one-way analysis of variance (Anova). A supra-additive or additive effect was seen when seven ovarian carcinoma cell lines were exposed to paclitaxel or docetaxel concomitantly with cisplatin. A supra-additive effect was found in four cell lines (UT-OC-3, UT-OC-4, UT-OC-5 and SK-OV-3) after simultaneous use of cisplatin with all docetaxel concentrations tested, and in two cell lines (UT-OC-4 and SK-OV-3) when cisplatin was used concomitantly with paclitaxel. A more pronounced supra-additive effect was seen with the combination of cisplatin and docetaxel. The degree of supra-additivity was dose dependent, with increasing synergy after a higher taxane dose. The data obtained in this study suggest that a supra-additive or additive effect can be achieved in ovarian carcinoma with the concomitant use of cisplatin and a taxane.

**Keywords** taxanes; cisplatin; interaction; chemosensitivity; ovarian carcinoma

The efficacy of paclitaxel and cisplatin combinations has been shown in two recent phase III trials (McGuire et al, 1996; Piccart et al, 1997), and this combination is currently widely used as the primary regimen for ovarian carcinoma. Docetaxel is another actively studied taxane. In vitro studies have shown that compared with paclitaxel, it has higher intracellular accumulation and binding to microtubules, as well as lower efflux and dissociation from microtubules (Riou et al, 1994; Lavelle et al, 1995). Phase I trials on docetaxel–cisplatin treatments are also ongoing. This combination appears promising in non-small-cell lung cancer and in a few other solid tumour types, including colorectal, head and neck, gastric and breast cancer (Burris et al, 1995). Preliminary results from phase II clinical trials on the use of docetaxel in advanced ovarian cancer have confirmed the data obtained from preclinical studies (Kaye et al, 1995). Cisplatin is the most effective single chemotherapeutic agent in the treatment of ovarian carcinoma (Thigpen et al, 1989; Advanced Ovarian Cancer Trialists Group, 1991). The role of docetaxel in the management of this disease will, therefore, depend on the cytotoxic effect achieved with docetaxel–cisplatin therapy.

We have recently studied the sensitivity of cisplatin, paclitaxel and docetaxel in seven epithelial ovarian carcinoma cell lines using a clonogenic assay. The $IC_{50}$ values of these drugs varied between 0.3 and 1.5 pM, 0.4 and 3.4 nM and 0.2 and 2.3 nM respectively (Engblom et al, 1996, 1997). On a molar basis, docetaxel was more cytotoxic than paclitaxel in six out of seven cell lines. The purpose of this study was to make a comparison between combinations of cisplatin–paclitaxel and of cisplatin–docetaxel in ovarian carcinoma cell lines, and to assess the types of interaction obtained. To our knowledge, comparative in vitro studies have not been previously published.

*Received 15 December 1997*
*Revised 9 June 1998*
*Accepted 11 June 1998*

*Correspondence to:* S Grènman, Department of Obstetrics and Gynecology, Turku University Central Hospital, FIN-20520 Turku, Finland

### MATERIALS AND METHODS

### Cell lines

Seven ovarian carcinoma cell lines were tested in this study. The cell lines used, their histological type, plating efficiencies (PE) and passages used are listed in Table 1. The SK-OV-3 and the CAOV-3 cell lines (Fogh et al, 1977; Untch et al, 1994) were obtained from the American Type Culture Collection (Rocville, MD, USA), and five cell lines (UT-OC-1, UT-OC-2, UT-OC-3, UT-OC-4 and UT-OC-5) have been established recently at the University of Turku by the author for correspondence. The UT-OC-5 cell line was derived from a metastatic omental tumour, whereas the other cell lines were established from primary tumours. The donor of the UT-OC-2 cell line had been treated with four courses of vincristine, doxorubicin and cyclophosphamide and radiotherapy for pulmonary metastases before the cell line was established from a primary tumour outside the radiation field. The donors of the UT-OC-4 and UT-OC-5 cell lines had received pelvic radiotherapy

**Table 1** Histological type, the passages used and the plating efficiency (PE) of the seven ovarian carcinoma cell lines and chemosensitivity of these cell lines to cisplatin, paclitaxel and docetaxel expressed as $IC_{50}$ values, corresponding to the drug concentration causing 50% inhibition of clonogenic survival

| Cell line | Histological type | Passages used | Plating efficiency | Cisplatin[a] $IC_{50}$ ± s.d. (pM) | Paclitaxel[a] $IC_{50}$ ± s.d. (nM) | Docetaxel[b] $IC_{50}$ ± s.d. (nM) |
|---|---|---|---|---|---|---|
| UT-OC-1 | Mucinous | 24–42 | 0.05–0.06 | 0.6 ± 0.2 | 1.4 ± 0.1 | 0.8 ± 0.1 |
| UT-OC-2 | Endometrioid | 10–24 | 0.06–0.09 | 0.3 ± 0.1 | 2.0 ± 0.2 | 2.3 ± 0.3 |
| UT-OC-3 | Serous | 20–37 | 0.09–0.2 | 0.7 ± 0.1 | 1.3 ± 0.1 | 1.0 ± 0.1 |
| UT-OC-4 | Endometrioid | 30–37 | 0.06–0.1 | 1.0 ± 0.2 | 1.0 ± 0.1 | 0.5 ± 0.1 |
| UT-OC-5 | Serous | 14–18 | 0.05–0.1 | 1.2 ± 0.2 | 1.4 ± 0.1 | 1.2 ± 0.2 |
| SK-OV-3 | Epithelial | 32–44 | 0.2–0.4 | 1.5 ± 0.1 | 3.4 ± 0.2 | 1.3 ± 0.2 |
| CAOV-3 | Papillary | 41–48 | 0.08–0.2 | 0.9 ± 0.1 | 0.4 ± 0.1 | 0.2 ± 0.1 |

[a]Engblom et al (1996); [b]Engblom et al (1997).

for cervical cancer 30 and 5 years before the diagnosis of ovarian carcinoma. The donors of the UT-OC-1 and UT-OC-3 cell lines had not received any cytotoxic therapy before the establishment of the cell lines.

### Cell culture

Before the experiments, the cells were kept in logarithmic growth in T25 culture flasks by passing weekly in Dulbecco's modified Eagle's minimal essential medium (DMEM) containing 2 mM L-glutamine, 1% non-essential amino acids, 100 U $ml^{-1}$ streptomycin, 100 U $ml^{-1}$ penicillin and 10% fetal bovine serum (FBS). Cells in mid-logarithmic growth (40–60% confluence) were used for the experiments and fed with fresh medium on the day before plating.

### Drug preparation

Cisplatin (Platinol) 0.5 mg $ml^{-1}$ was diluted with growth medium to get a stock solution of 100 μg $ml^{-1}$. Final cisplatin dilutions of 0.05–0.6 μg $ml^{-1}$ were used, and new stock solutions were made for each experiment. Paclitaxel (Taxol, kindly provided by Bristol-Myers Squibb) was initially dissolved in 0.9% sodium chloride to get a solution of 0.1 mM. Stock solutions were prepared in Ham's F-12 medium containing 10% FBS to obtain a solution of 100 nM, and stored at –40°C. Final dilutions of 0.4–5 nM paclitaxel were used for the experiments. Docetaxel (Taxotere, 807.9 mg, kindly provided by Rhone-Poulenc Rorer) was diluted in 1 ml of ethanol to obtain a stock solution of 0.1 mM and stored at –40°C. These solutions were further diluted in sterile water to obtain a solution of 100 nM immediately before each experiment. Final dilutions of 0.3–4 nM docetaxel were used for the experiments. We have previously studied the sensitivity of cisplatin, paclitaxel and docetaxel in these cell lines (Engblom et al, 1996, 1997). The $IC_{50}$ values obtained in these experiments are given in Table 1 and were used as the basis of drug concentrations used in this study. The paclitaxel concentrations used in this study corresponded to 25–100% of the $IC_{50}$ values of the cell lines. The docetaxel concentration varied from 25% to 150% of the $IC_{50}$ value.

### Clonogenic assay

The 96-well plate clonogenic assay based on limiting dilutions was used. The assay has been described earlier in detail (Grènman et al, 1989; Rantanen et al, 1994). The cells were harvested with trypsin-EDTA to obtain a single-cell suspension, counted and diluted in Ham's F-12 medium containing 15% FBS. The number of cells plated per well was adjusted according to the PE of the cell line. The desired concentrations of paclitaxel or docetaxel were added in a stock solution containing 4167 cells $ml^{-1}$, and diluted in 25 ml of growth medium. A concentration of two cells per well is achieved by applying 100 μl of this stock solution to each well of the 96-well plate. The desired cisplatin concentrations along with the same paclitaxel or docetaxel concentration as on the day before were added in 100 μl of growth medium after the plates had been incubated for 24 h. All the drugs were allowed to stay on the plates throughout the whole incubation period. The plates were incubated at 37°C with 5% carbon dioxide for 4 weeks, after which the number of wells containing coherent, living colonies, consisting of 32 cells or more, was counted using an inverted phase-contrast microscope.

### Data analysis

PE was calculated by the formula PE = –ln (number of negative wells/total number of wells)/number of cells plated per well (Thilly et al, 1980). Fraction survival data were fitted to the linear quadratic model, $F = \exp[-(\alpha D+\beta D^2)]$ and a microcomputer program was used to obtain the area under the curve (AUC) by numerical integration. The simultaneous effects of cisplatin and paclitaxel or docetaxel were determined as the ratio between the AUC for cisplatin plus paclitaxel or docetaxel, divided by the AUC for cisplatin alone. This AUC ratio was compared with the surviving fraction (SF) after the indicated dose of paclitaxel or docetaxel alone. Comparison of the AUC ratio and the SF value was made using the Student's *t*-test. The influence of the taxane concentration on the amount of additive or supra-additive cytotoxic effect was tested by one-way analysis of variance (ANOVA). The schedule of the drug administration is important and the achieved growth inhibition can vary from a subadditive to a supra-additive effect. To describe the type of interaction, we have used the term additive of the sum of individual effects. The term supra-additive is used if the combined effect exceeds the sum of individual effects. Some authors use the term synergy, which we have interpreted here as supra-additivity.

### RESULTS

Cisplatin and taxanes had either an additive or supra-additive growth inhibitory effect in all cell lines studied. The type and magnitude of growth inhibition varied between individual cell lines. In most of the cell lines, higher taxane concentrations

© Cancer Research Campaign 1999

**Table 2** Effects of paclitaxel on clonogenic survival of seven ovarian carcinoma cell lines. Paclitaxel was used as a single agent and concomitantly with cisplatin

| Cell line | Paclitaxel dose (nM) | Cisplatin dose (μg ml⁻¹) | $S^{a}_{paclitaxel}$ | AUC ratio[b] | *P*-value |
|---|---|---|---|---|---|
| UT-OC-1 | 0.6 | 0.05–0.15 | 0.91 ± 0.07 | 0.89 ± 0.03 | 0.45 (A) |
| | 0.8 | | 0.78 ± 0.04 | 0.80 ± 0.03 | 0.30 (A) |
| | 1.0 | | 0.68 ± 0.03 | 0.59 ± 0.03 | 0.0003 (SA) |
| UT-OC-2 | 0.8 | 0.01–0.1 | 0.90 ± 0.10 | 0.87 ± 0.08 | 0.69 (A) |
| | 1.0 | | 0.88 ± 0.06 | 0.88 ± 0.08 | 0.97 (A) |
| | 2.0 | | 0.72 ± 0.05 | 0.72 ± 0.08 | 0.91 (A) |
| UT-OC-3 | 0.6 | 0.05–0.4 | 0.94 ± 0.02 | 1.01 ± 0.06 | 0.58 (A) |
| | 0.8 | | 0.77 ± 0.11 | 0.86 ± 0.12 | 0.49 (A) |
| | 1.0 | | 0.67 ± 0.11 | 0.70 ± 0.08 | 0.68 (A) |
| UT-OC-4 | 0.4 | 0.2–0.6 | 1.00 ± 0.04 | 0.92 ± 0.05 | 0.0046 (SA) |
| | 0.6 | | 0.93 ± 0.02 | 0.84 ± 0.05 | 0.0021 (SA) |
| | 0.8 | | 0.63 ± 0.02 | 0.33 ± 0.05 | 0.0001 (SA) |
| UT-OC-5 | 0.6 | 0.2–0.5 | 0.97 ± 0.02 | 0.85 ± 0.05 | 0.071 (A) |
| | 0.8 | | 0.88 ± 0.04 | 0.63 ± 0.05 | 0.011 (SA) |
| | 1.0 | | 0.68 ± 0.08 | 0.42 ± 0.06 | 0.056 (A) |
| SK-OV-3 | 1.5 | 0.3–0.6 | 0.95 ± 0.05 | 0.95 ± 0.03 | 0.021 (SA) |
| | 2.0 | | 0.91 ± 0.04 | 0.76 ± 0.03 | 0.0008 (SA) |
| | 3.0 | | 0.66 ± 0.03 | 0.51 ± 0.06 | 0.036 (SA) |
| CAOV-3 | 0.1 | 0.1–0.5 | 0.96 ± 0.08 | 0.91 ± 0.07 | 0.11 (A) |
| | 0.2 | | 0.93 ± 0.06 | 0.83 ± 0.12 | 0.25 (A) |
| | 0.3 | | 0.81 ± 0.03 | 0.68 ± 0.05 | 0.0065 (SA) |

[a]Clonogenic survival after the indicated paclitaxel dose; [b]the ratio between the AUC for cisplatin plus paclitaxel, divided by the AUC for cisplatin alone. *P*-values were calculated using the Student's *t*-test. A, additive effect; SA, supra-additive effect.

**Table 3** Effects of docetaxel on clonogenic survival of seven ovarian carcinoma cell lines. Docetaxel was used as a single agent and concomitantly with cisplatin

| Cell line | Docetaxel dose (nM) | Cisplatin dose (μg ml⁻¹) | $S^{a}_{docetaxel}$ | AUC ratio[b] | *P*-value |
|---|---|---|---|---|---|
| UT-OC-1 | 0.2 | 0.005–0.15 | 1.00 ± 0.03 | 0.96 ± 0.05 | 0.15 (A) |
| | 0.5 | | 0.89 ± 0.03 | 0.81 ± 0.05 | 0.039 (SA) |
| | 0.8 | | 0.65 ± 0.06 | 0.57 ± 0.04 | 0.019 (SA) |
| UT-OC-2 | 0.8 | 0.01–0.1 | 0.79 ± 0.07 | 0.97 ± 0.05 | 0.0087 (SA) |
| | 1.0 | | 0.73 ± 0.09 | 0.74 ± 0.07 | 0.19 (A) |
| | 1.5 | | 0.68 ± 0.03 | 0.68 ± 0.05 | 0.87 (A) |
| UT-OC-3 | 0.5 | 0.05–0.4 | 0.77 ± 0.04 | 0.71 ± 0.02 | 0.021 (SA) |
| | 0.8 | | 0.72 ± 0.05 | 0.53 ± 0.03 | 0.0001 (SA) |
| | 1.0 | | 0.68 ± 0.04 | 0.45 ± 0.03 | 0.0001 (SA) |
| UT-OC-4 | 0.3 | 0.2–0.6 | 0.80 ± 0.03 | 0.71 ± 0.04 | 0.0005 (SA) |
| | 0.4 | | 0.64 ± 0.05 | 0.48 ± 0.08 | 0.0001 (SA) |
| | 0.5 | | 0.53 ± 0.08 | 0.37 ± 0.07 | 0.0001 (SA) |
| UT-OC-5 | 0.4 | 0.2–0.5 | 0.83 ± 0.03 | 0.74 ± 0.03 | 0.0030 (SA) |
| | 0.6 | | 0.75 ± 0.04 | 0.64 ± 0.03 | 0.0006 (SA) |
| | 0.8 | | 0.64 ± 0.04 | 0.51 ± 0.003 | 0.0017 (SA) |
| SK-OV-3 | 0.8 | 0.3–0.6 | 0.94 ± 0.04 | 0.80 ± 0.04 | 0.0002 (SA) |
| | 1.0 | | 0.90 ± 0.04 | 0.75 ± 0.05 | 0.0002 (SA) |
| | 1.3 | | 0.81 ± 0.05 | 0.57 ± 0.07 | 0.0001 (SA) |
| CAOV-3 | 0.1 | 0.1–0.5 | 0.95 ± 0.04 | 0.92 ± 0.07 | 0.10 (A) |
| | 0.2 | | 0.92 ± 0.04 | 0.76 ± 0.12 | 0.0097 (SA) |
| | 0.3 | | 0.69 ± 0.05 | 0.50 ± 0.08 | 0.015 (SA) |

[a]Clonogenic survival after the indicated docetaxel dose; [b]ratio between the AUC for cisplatin plus docetaxel divided by the AUC for cisplatin alone. *P*-values were calculated using the Student's *t*-test. A, additive effect; SA, supra-additive effect.

increased the extent of supra-additive effect. In some cell lines, lower drug concentrations caused an additive effect, whereas higher concentrations were supra-additive. Furthermore, on a molar basis, docetaxel–cisplatin combinations had more pronounced cytotoxic effects than paclitaxel–cisplatin combinations. A supra-additive effect was seen more frequently with a cisplatin–docetaxel combination than with a cisplatin–paclitaxel combination (Tables 2 and 3).

The type of interaction after paclitaxel and cisplatin and the statistical significance of supra-additivity is presented in Table 2. Dose dependency of the magnitude of the interaction is presented in Table 4. All paclitaxel concentrations used concomitantly with

 *© Cancer Research Campaign 1999*

**Table 4** The dose dependence of additive and supra-additive cytotoxic effect. The influence of the concentration was tested by one-way analysis of variance (Anova) and the *P*-values after cisplatin plus paclitaxel and cisplatin plus docetaxel are listed below. A statistically significant direct correlation between increasing the taxane dose and the amount of synergy is found when *P* is < 0.05

| Cell line | Cisplatin plus paclitaxel | Cisplatin plus docetaxel |
|---|---|---|
| UT-OC-1 | 0.0032 | 0.53 |
| UT-OC-2 | 0.88 | [a] |
| UT-OC-3 | 0.84 | 0.0001 |
| UT-OC-4 | 0.0001 | 0.0050 |
| UT-OC-5 | 0.24 | 0.24 |
| SK-OV-3 | 0.015 | 0.0004 |
| CAOV-3 | 0.59 | 0.027 |

[a]UT-OC-2 cell line was an exception because the combined effect was supra-additive with the lowest docetaxel dose and additive with the two higher doses.

cisplatin caused a supra-additive growth inhibitory effect in the UT-OC-4 and SK-OV-3 cell lines. Additive effect was found with all tested paclitaxel concentrations in UT-OC-2 and UT-OC-3 cells. In CAOV-3 cells, the combined effect was additive (*P*-values 0.11 and 0.25) when cisplatin was added to 0.1 or 0.2 nM paclitaxel, which corresponds to 50% or 100% of the previously determined $IC_{50}$ concentration. In contrast, 0.3 nM paclitaxel caused a clear supra-additive (*P* = 0.0065) effect. In UT-OC-1 cells, an additive growth inhibitory effect (*P*-values 0.45 and 0.30) was noticed with 0.6 and 0.8 nM paclitaxel, corresponding to 43% and 57% of the $IC_{50}$ dose (Table 2). The UT-OC-1 cells showed a clear supra-additive effect when 1.0 nM of paclitaxel was combined with cisplatin. In cell lines showing supra-additivity with lower paclitaxel doses, the increasing paclitaxel dose resulted in increased supra-additivity. In the UT-OC-1, UT-OC-4 and SK-OV-3 cell lines, the degree of supra-additivity was found to be directly correlated to the dose of paclitaxel and this correlation was statistically significant (Table 4). The fitted survival curves of the seven ovarian carcinoma cell lines with three various paclitaxel doses combined with cisplatin are shown in Figure 1.

The type of interaction after docetaxel and cisplatin and the statistical significance of supra-additivity is shown in Table 3, and the dose dependency of interaction is presented in Table 4. In four cell lines (SK-OV-3, UT-OC-3, UT-OC-4 and UT-OC-5), a supra-additive effect was found after simultaneous use of cisplatin with all tested docetaxel concentrations. The lowest docetaxel dose used in the CAOV-3 and UT-OC-1 cells, corresponding to 50% and 25%, respectively, of the $IC_{50}$ doses of the cell lines, caused a pure additive effect (*P*-values 0.10 and 0.15), whereas with a higher docetaxel dose supra-additivity was found. The UT-OC-2 cell line was an exception because the combined effect was supra-additive with the lowest docetaxel dose, and additive with the two higher doses (Table 3). The degree of supra-additivity was dose dependent in SK-OV-3, UT-OC-3 and UT-OC-4 cell lines. Increasing the docetaxel dose resulted in a clearer supra-additive effect. The same phenomenon was noticed also in CAOV-3 and UT-OC-1 cells, though in these cell lines the lowest docetaxel dose caused a purely additive effect (Table 3). The fitted survival curves of the seven cell lines after concomitant exposure to docetaxel and cisplatin are shown Figure 1.

### DISCUSSION

In this study, we demonstrated a supra-additive or additive growth-inhibitory effect when human ovarian carcinoma cells were exposed to paclitaxel or docetaxel concomitantly with cisplatin. This effect was found to be dose dependent with the combination of paclitaxel and cisplatin in three cell lines and with the combination of docetaxel and cisplatin in four out of seven cell lines (Table 4). The UT-OC-2 cell line was an exception; simultaneous docetaxel and cisplatin caused a clear supra-additive effect with the lowest docetaxel dose and an additive effect with the two higher doses. In our previous study, we have shown that clonogenic cell survival after paclitaxel or docetaxel exposure clearly correlated in six out of seven ovarian carcinoma cell lines (Engblom et al, 1997). The only exception was the UT-OC-2 cell line. This result was consistent in repeated experiments. On a molar basis, all seven ovarian cell lines showed more pronounced supra-additivity with the combination of docetaxel and cisplatin compared with paclitaxel and cisplatin.

The effects of paclitaxel in combination with cisplatin were initially reported by Citardi and colleagues in 1990 in mouse leukaemia L1210 cells. They demonstrated the superiority of paclitaxel given before cisplatin compared with other regimens (Citardi et al, 1990). In ovarian cancer cell lines, the decrease of cell viability was significantly greater with the combination of paclitaxel and cisplatin compared with exposure to a single drug (Untch et al, 1994). With human ovarian carcinoma cells, additive or supra-additive effect was found when the cells were exposed to paclitaxel before cisplatin. Conversely, if cisplatin was given first, antagonism was observed (Parker et al, 1993; Jekunen et al, 1994; Kiyozuka et al, 1995). In the current experiments, an additive or supra-additive inhibitory effect was seen in all cell lines when the taxane were administered concomitantly with cisplatin. This is in line with previously published reports showing an additive (Saunders et al, 1992; Jekunen et al, 1994) or supra-additive (Parker et al, 1993) effect with the cisplatin–paclitaxel combination in ovarian cell lines. The growth inhibitory effect of docetaxel combined with cytotoxic agents has not been studied as widely as that of paclitaxel. In a study with human breast carcinoma cells, an additive or supra-additive effect was noticed after cells pretreated with edatrexate were treated with docetaxel. However, antagonism was evident when the schedule was reversed (Chou et al, 1996). In the present study, we demonstrated a supra-additive or additive growth inhibitory effect when docetaxel was given concomitantly with cisplatin. Moreover, on a molar basis, this combination was more effective than the combination of paclitaxel and cisplatin.

The type and degree of the growth inhibitory effect varied with different doses of the taxanes. Increasing paclitaxel doses resulted in increasing supra-additivity in three out of seven cell lines. The same kind of dose-dependent interaction was found in the breast cancer cell lines (Koechli et al, 1993). The dose dependency of the cisplatin–docetaxel combination was even more pronounced because the degree of supra-additivity was dose dependent in three cell lines. In an additional two cell lines, the lowest docetaxel dose had an additive effect and higher doses had a supra-additive growth inhibitory effect.

It has been demonstrated in several studies that on a molar basis docetaxel is more potent than paclitaxel as a single drug (Kelland et al, 1992; Riou et al, 1992; Hill et al, 1994; Engblom et al, 1997). In the present study, a greater supra-additive effect was achieved with the combination of docetaxel and cisplatin compared with the

© Cancer Research Campaign 1999

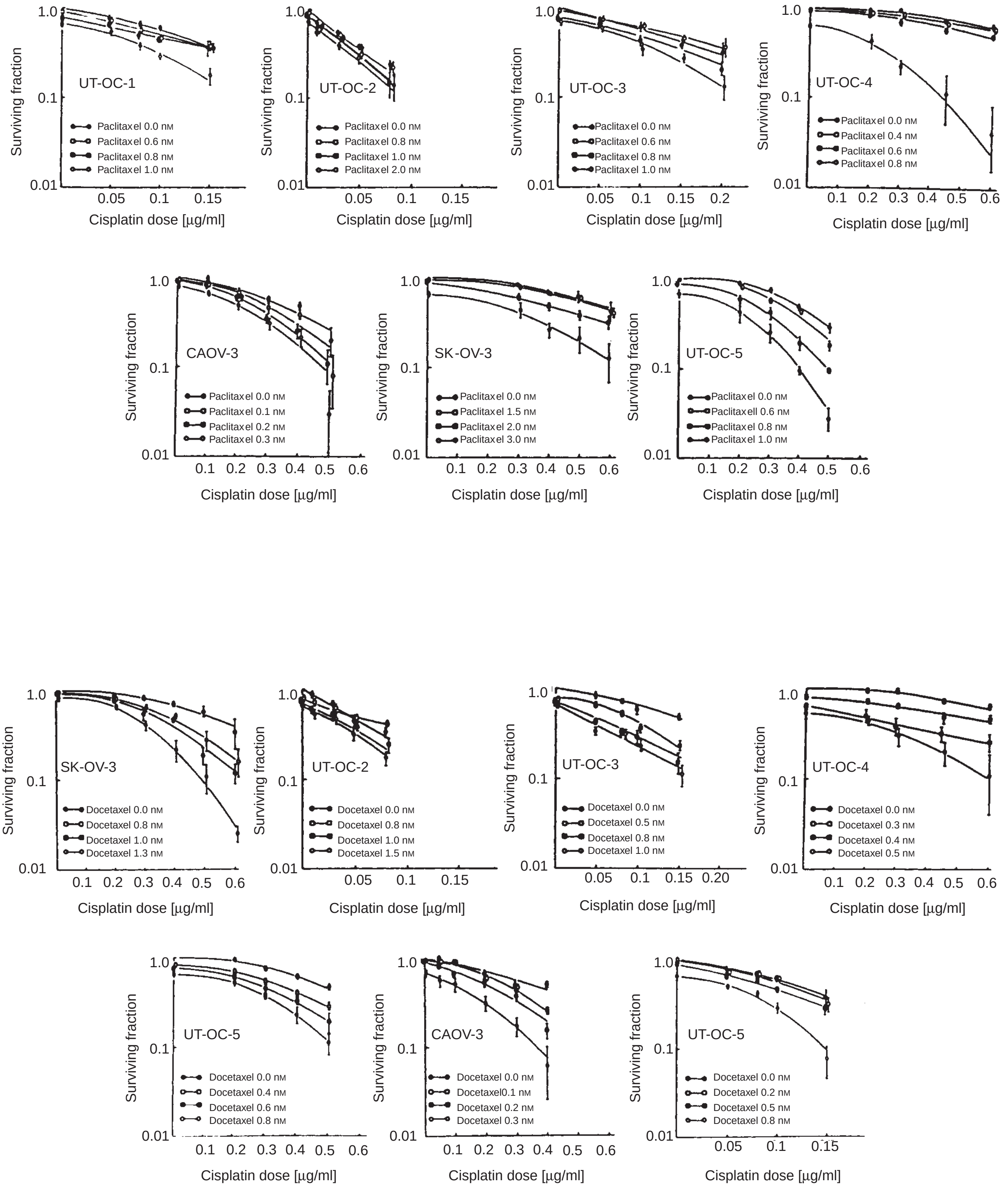

**Figure 1** Effects of simultaneous use of cisplatin and paclitaxel or docetaxel. Fitted cisplatin curves for the seven ovarian carcinoma cell lines without paclitaxel or docetaxel and combined with the desired taxane doses. The results are given as the average of the actual data points and the bars represent one standard deviation

 *© Cancer Research Campaign 1999*

combination of paclitaxel and cisplatin. Studies evaluating the mechanism of action of these two taxanes have shown that, in comparison with paclitaxel, docetaxel is slightly more active as a tubulin assembly promoter and microtubule stabilizer, and approximately twofold more potent as an inhibitor of microtubule depolymerization (Gueritte-Voegelein et al, 1991). Furthermore, the effective affinity of docetaxel for the microtubule binding site is 1.9-fold greater than that of paclitaxel (Diaz et al, 1993). These differences in mechanism of action may explain the differences in the cytotoxic effect achieved with concomitant use of cisplatin and these two taxanes.

Peak plasma concentrations achieved with a 24-h paclitaxel infusion have ranged from 0.23 to 0.43 μM, and with a 3-h infusion from 2.5 to 4.3 μM (Huizing et al, 1993). After a 1-h infusion, the peak plasma concentration for docetaxel has been 4.46 μM (Hino et al, 1995), and for cisplatin 2.5 μg $ml^{-1}$ (Gullo et al, 1980). The current experiments were performed using paclitaxel concentrations of 0.1–3 nM, docetaxel doses of 0.1–1.5 nM and cisplatin doses of 0.01–0.6 μg $ml^{-1}$, which were clearly below the peak plasma concentrations achieved for these drugs. In vitro, the duration of both paclitaxel (Rowinsky et al, 1988; Arbuck et al, 1993; Lopes et al, 1993; Georgiadis et al, 1994) and docetaxel (Hill et al, 1994) exposure has a great impact on the growth-inhibitory effect of the drug.

In fact, in studies combining taxanes and radiation, increasing the time of exposure has been reported to be more important than increasing the drug concentration (Schiff et al, 1995). In the present study, the time of exposure was long and was kept constant, and the interaction of cisplatin and taxanes was studied as the function of drug concentrations.

The efficacy of the cisplatin–paclitaxel combination has been demonstrated in clinical use. Incorporating paclitaxel into first-line therapy has improved the survival in stage III and stage IV ovarian carcinoma (McGuire et al, 1996; Piccart et al, 1997). The therapeutic effect of docetaxel–cisplatin combination is under investigation. The results of the present study indicate that on a molar basis the combination of docetaxel–cisplatin is more cytotoxic than the combination of paclitaxel–cisplatin. If the toxicity profile of the docetaxel–cisplatin combination is acceptable, a randomized trial comparing the two taxane–cisplatin combinations is warranted.

### ACKNOWLEDGEMENTS

This study was supported by grants from the Southernwestern Division of the Finnish Cancer Society and Turku University Foundation. We want to express our gratitude to Mrs Marita Potila for her excellent technical assistance in performing the experiments. Cisplatin was generously provided by Orion Corporation Farmos, Turku, Finland, paclitaxel by Bristol-Myers Squibb, and docetaxel by Rhone-Poulenc Rorer.

### REFERENCES

Advanced Ovarian Cancer Trialists Group (1991) Chemotherapy in advanced ovarian cancer: an overview of randomised clinical trials. *Br Med J* **303**: 884–893

Arbuck SG, Canetta R and Onetto N (1993) Current dosage and scheduling issues in the development of paclitaxel (TAXOL). *Semin Oncol* **4** (suppl. 3): 31–39

Burris HA, Fields S and Peacock N (1995) Docetaxel (Taxotere) in combination: a step forward. *Semin Oncol* **22**: 35–40

Chou TC, Otter GM and Sirotnak FM (1996) Schedule-dependent synergism of taxol or taxotere with edatrexate against human breast cancer cells in vitro. *Cancer Chemother Pharmacol* **37**: 222–228

Citardi MJ, Rowinsky EK, Schaefer KL and Donehower RC (1990) Sequence-dependent cytotoxicity between cisplatin and the antimicrotubule agents taxol and vincristine. *Proc Am Assoc Cancer Res* **34**: 2431

Diaz JF, Menendez M and Andreau JM (1993) Assembly of purified GDP-tubulin into microtubules induced by RP 56976 and paclitaxel: reversibility, ligand stoichiometry and competition. *Biochemistry* **32**: 2747–2755

Engblom P, Rantanen V, Kulmala J and Grènman S (1996) Paclitaxel and cisplatin sensitivity of ovarian carcinoma cell lines tested with the 96-well plate clonogenic assay. *Anticancer Res* **16**: 1743–1748

Engblom P, Rantanen V, Kulmala J, Heiskanen J and Grenman S (1997) Taxane sensitivity of ovarian carcinoma in vitro. *Anticancer Res* **17**: 2475–2480

Fogh J, Wright WC and Loveless JD (1977) Absence of Hela cell contamination on 169 cell lines derived from human tumours. *J Natl Cancer Inst* **58**: 209–214

Georgiadis MS, Russell E and Johnson BE (1994) Prolonging the exposure of human lung cancer cell lines to paclitaxel increases the cytotoxicity. *Proc Int Assoc Study Lung Cancer* **11**: 95

Grènman R, Burk D, Virolainen E, Buick RN, Church J, Schwartz DR and Carey TE (1989) Clonogenic cell assay for anchorage-dependent squamous carcinoma cell lines using limiting dilution. *Int J Cancer* **44**: 131–136

Gueritte-Voegelein F, Guenard D, Lavelle F, Le Goff MT, Mangatal L and Potier P (1991) Relationships between the structure of taxol analogues and their antimitotic activity. *J Med Chem* **34**: 992–998

Gullo JJ, Litterst AR, McGuire WP, Sikic B, Hoth J and Wooley PA (1980) Pharmacokinetics and protein-binding of *cis*-dichlorodiamineplatinum (11) administered as a one hour or as a twenty-four hour infusion. *Cancer Chemother Pharmacol* **5**: 21–26

Hill BT, Whelan RDH, Shellard SA, McClean S and Hosking LK (1994) Differential cytotoxic effects of docetaxel in a range of mammalian tumour cell lines and certain drug resistant sublines in vitro. *Invest New Drugs* **12**: 169–182

Hino M, Kobayashi K, Hayashihara K, Aoyama A, Shibuya M and Kudo S (1995) In vitro combination effect of docetaxel (RP 56976) with vinca alkaloids on cancer cell line. *Proc Am Assoc Cancer Res* **36**: 2995

Huizing MT, Keung ACF and Rosing H (1993) Pharmacokinetics of paclitaxel and metabolites in a randomised comparative study in platinum-pre-treated ovarian cancer patients. *J Clin Oncol* **11**: 2127–2135

Jekunen A, Christen R, Shalinsky D and Howell SB (1994) Synergistic interaction between cisplatin and taxol in human ovarian carcinoma in vitro. *Br J Cancer* **69**: 299–306

Kaye SB, Piccart M, Aapro M and Kavanagh JJ (1995) Docetaxel in advanced ovarian cancer: preliminary results from three phase II trials. *Eur J Cancer* **31A**: 14–17

Kelland LR and Abel G (1992) Comparative in vitro cytotoxicity of taxol and taxotere against cisplatin-sensitive and resistant human ovarian carcinoma cell lines. *Cancer Chemother Pharmacol* **30**: 444–450

Kiyozuka Y, Nishimura H, Nakashima A, Imamura K, Ota S, Yakushiji OM, Imai S and Morimatsu M (1995) In vitro cytotoxicity on ovarian cancer of low-dose continuous exposure to microtubule inhibitors (paclitaxel/taxotere) combined with cisplatin. *Oncol Rep* **2**: 517–523

Koechli O, Sevin B-U, Perras J, Chao Chou T, Angioli R, Steren A, Untch M and Averette HE (1993) Characteristics of the combination paclitaxel plus doxorubicin in breast cancer cell lines analyzed with the ATP-cell viability assay. *Breast Cancer Res Treatment* **28**: 21–27

Lavelle F, Bissery M-C, Combeau C, Riou JF, Vrignaud P and Andrè S (1995) Preclinical evaluation of docetaxel (Taxotere). *Semin Oncol* **22**: 3–16

Lopes NM, Adams EG and Pitts TW (1993) Cell kill kinetics and cell cycle effects of Taxol on human and hamster ovarian cell lines. *Cancer Chemother Pharmacol* **32**: 235–242

McGuire WP, Hoskins WJ, Brady MF, Kucera PR, Partridge EE, Look KY, Clarke-Pearson DL and Davidson M (1996) Cyclophosphamide and cisplatin compared with paclitaxel and cisplatin in patients with stage III and stage IV ovarian cancer. *N Engl J Med* **334**: 1–6

Parker R, Lee KB and Dabholkar M (1993) Influence of taxol: cisplatin sequencing on cisplatin-DNA adduct repair in human ovarian cancer cells. *Proc Am Assoc Cancer Res* **34**: 2122

Piccart MJ, Bertelsen K and Stuart G (1997) Is cisplatin-paclitaxel (P-T) the standard in first-line treatment of advanced ovarian cancer (Ov Ca)? The EORTC-GCCG, NOVOCA, NCI-C and Scottish Intergroup experience. *Proc Am Soc Clin Oncol* **16**: 352a

Rantanen V, Grènman S, Kulmala J and Grènman R (1994) Comparative evaluation of cisplatin and carboplatin sensitivity in endometrial adenocarcinoma cell lines. *Br J Cancer* **69**: 482–486

© Cancer Research Campaign 1999

Riou JF, Naudin A and Lavelle F (1992) Effects of taxotere on murine and human tumour cell lines. *Biochem Biophys Res Commun* **187**: 164–170

Riou JF, Petingenet O, Combeau C and Lavelle F (1994) Cellular uptake and efflux of docetaxel (Taxotere) and paclitaxel (Taxol) in P388 cell line. *Proc Am Assoc Cancer Res* **35**: 384

Rowinsky EK, Donehower RC and Jones RJ (1988) Microtubule changes and cytotoxicity in leukaemia cell lines treated with Taxol. *Cancer Res* **48**: 4093–4100

Saunders DE, Christensen C and LoRusso PM (1992) Inhibition of ovarian carcinoma cells by taxol combined with vitamin D and adriamycin. *Proc Am Assoc Cancer Res* **33**: 2641

Schiff PB, Gubits R and Kashimawo (1995) Paclitaxel with ionising radiation. In *Paclitaxel in Cancer Treatment*, McGuire WP and Rowinsky EK (eds), pp. 81–90. Marcel Dekker: New York

Thigpen JT, Blessing JA, Vance RB and Lambuth BW (1989) Chemotherapy in ovarian carcinoma: present role and future prospects. *Semin Oncol* **16**: 58–65

Thilly WG, Deluca JG, Furth EE, Hoppe H, Kaden DA, Krolenski JJ, Liber HL, Skopek TR, Laslapikoff SA, Tizard RJ and Penman BW (1980) Gene-locus mutation assays in diploid human lymphoblast lines. In *Chemical mutagens*. de Serpes FJ and Hollander A (eds), pp. 331–364. Plenum: New York

Untch M, Sevin B-U, Perras JP, Angioli DR, Untch A, Hightower RD, Koechli O and Averette HE (1994) Evaluation of paclitaxel (Taxol), cisplatin and the combination paclitaxel-cisplatin in ovarian cancer in vitro with the ATP-cell viability assay. *Gynecol Oncol* **53**: 44–49

*© Cancer Research Campaign 1999*
